# TRP Channels Expression Profile in Human End-Stage Heart Failure

**DOI:** 10.3390/medicina55070380

**Published:** 2019-07-16

**Authors:** Martin Dragún, Andrea Gažová, Ján Kyselovič, Michal Hulman, Marek Máťuš

**Affiliations:** 1 Department of Pharmacology and Toxicology, Faculty of Pharmacy, Comenius University in Bratislava, Odbojarov 10, 83232 Bratislava, Slovakia; 2 Institute of Pharmacology and Clinical Pharmacology, Faculty of Medicine, Comenius University in Bratislava, Spitalska 24, 81372 Bratislava, Slovakia; 3 5th Department of Internal Medicine, University Hospital Bratislava—Ružinov, Ruzinovska 6, 82606 Bratislava, Slovakia; 4 Clinic of cardiac surgery, National Institute for Cardiovascular Diseases, Pod Krasnou Horkov 1, 83348 Bratislava, Slovakia

**Keywords:** heart failure, human, TRP channels, calcium signaling

## Abstract

*Objectives*: Many studies indicate the involvement of transient receptor potential (TRP) channels in the development of heart hypertrophy. However, the data is often conflicted and has originated in animal models. Here, we provide systematic analysis of TRP channels expression in human failing myocardium. *Methods and results*: Left-ventricular tissue samples were isolated from explanted hearts of NYHA III-IV patients undergoing heart transplants (n = 43). Quantitative real-time PCR was performed to assess the mRNA levels of TRPC, TRPM and TRPV channels. Analysis of functional, clinical and biochemical data was used to confirm an end-stage heart failure diagnosis. Compared to myocardium samples from healthy donor hearts (n = 5), we detected a distinct increase in the expression of TRPC1, TRPC5, TRPM4 and TRPM7, and decreased expression of TRPC4 and TRPV2. These changes were not dependent on gender, clinical or biochemical parameters, nor functional parameters of the heart. We detected, however, a significant correlation of TRPC1 and MEF2c expression. *Conclusions*: The end-stage heart failure displays distinct expressional changes of TRP channels. Our findings provide a systematic description of TRP channel expression in human heart failure. The results highlight the complex interplay between TRP channels and the need for deeper analysis of early stages of hypertrophy and heart failure development.

## 1. Introduction

Heart failure (HF) is a culmination of different pathophysiological conditions on heart muscle which cause progressive worsening of heart function [1]. HF is usually preceded by chronic cardiac stress that could be the result of a cardiac injury (e.g., myocardial infarct, hypertension, valvular heart disease) and leads to structural changes, such as left ventricle hypertrophy, to maintain physiologic heart output [2,3]. Functionally, HF is preceded by impaired calcium homeostasis—Contraction and Ca^2+^ transients are markedly prolonged and restoration of low diastolic Ca^2+^ concentrations is impaired [4]. Increased Ca^2+^ levels during diastole can be related to depressed protein expression levels of sarco/endoplasmic reticulum Ca^2+^-ATPase (SERCA) or decreased rates of sarcoplasmic reticulum (SR) Ca^2+^ uptake [5]. After depletion of internal Ca^2+^ stores, the store-operated calcium entry (SOCE) is activated [6]. Multiple proteins and channels are involved in SOCE, e.g., STIM proteins, Orai channels or channels from the transient receptor potential (TRP) family.

TRP channels are a family of non-voltage dependent calcium-permeable ion channels first discovered in Drosophila [7]. The mammalian TRP family represents a big group of 28 cation channels divided according their structural homology into six subfamilies: TRP canonical (TRPC), TRP melastin (TRPM), TRP vanniloid (TRPV), TRP ankyrin (TRPA), TRP mucolipin (TRPML) TRP polycystin (TRPP) [8]. TRP can be activated by various stimuli, such as thermally-activated channels (TRPV, TRPM or TRPA) or TRPC activated by phospholipase C (PLC) [9]. There are also differences inside the groups in regulation of the channel activity e.g., TRPC subfamily, where TRPC3 and TRPC6 can be activated by stretch [9], TRPC1 and TRPC3 by SOCE [10], TRPC4 and TRPC5 are regulated by G protein-coupled receptor [11] and TRPC6 and TRPC7 are regulated by Ca^2+^ in multiple ways [12].

The physiological function of TRP channels in the heart is still under debate. Inhibition of TRP channels did not cause any defects under physiological conditions, but they seem to play a critical role in pathophysiology of numerous heart diseases [13]. TRP channels most often linked with hypertrophy and HF are from the TRPC subfamily. There are multiple animal and cell culture models of hypertrophy and HF confirming their role in these pathologies: knockout of *Trpc* genes or use of TRPC inhibitors prevents hypertrophy and HF development, and their increased expressions was detected after neuroendocrine agonist infusion (e.g., endothelin-1, phenylephrine and angiotensin II) and/or pressure overload stimulation [13,14]. It appears that they act through activation of calcineurin/nuclear factor of activated T cells (NFAT) signaling pathway, which leads to the activation of the pro-hypertrophic gene expression. Additionally, it has been shown that calcineurin/NFAT cascade can positively regulate the activity of TRPC channels [15,16].

In addition to TRPC channels, some members of TRPM and TRPV channels are also involved in hypertrophy and HF development. Inhibition of TRPV2 accumulation at sarcolemma improves the survival of animals with HF, prevents fibrosis and ventricular dilation and decreases the activation of calmodulin-dependent protein kinase II [17]. Moreover, disruption of the *Trpv1* gene can prevent the progression of cardiac hypertrophy [18].

However, the data on the role of TRP channels in the human HF is less complete or anecdotal. To our knowledge, only three studies have touched this topic in systematic manner [15,19,20]. The aim of this study was to investigate the gene expression of channels from the TRPC, TRPM and TRPV families in human heart and their possible expression changes in human HF compared to a non-failing healthy myocardium.

## 2. Materials and Methods

### 2.1. Human Heart Samples

For this study, 43 patients were chosen who were diagnosed with heart failure in NYHA III-IV class (HF group). These patients were indicated for heart transplant surgery and underwent the surgery in National Institute for Cardiovascular Diseases from August 2009 to June 2013. The samples of heart tissue were isolated from explanted left ventricle without epicardial fat, as a cross-section of the whole wall of the ventricle. Tissue samples of non-failing controls (CON; n = 5) were obtained from donor hearts that were prepared for transplantation, but the surgery was not performed. Clinical and biochemical data were obtained from patient documentation and were not older than 3–6 months before transplantation, except the heart weight which was measured after the surgery. Because the clinical data was not available from all patients, the number of data points for statistical evaluation can differ across the data groups and is noted in respective tables. The whole project and experimental procedures were approved by the Ethics committee of the National Institute for Cardiovascular Diseases in Bratislava, Slovak republic (No. IK-NP-0021-24/1426/14 and NUSCH EK 126/180509 from 11 November 2008). The study conformed to the principles outlined in the Declaration of Helsinki, all patients (or their legal representatives) were informed about and gave informed consent prior to participation in this project.

### 2.2. RNA Isolation and Real-Time Polymerase Chain Reaction

Total RNA was isolated from left ventricle samples using Tri-Reagent^®^ (Sigma-Aldrich, St. Louis, MO, USA) according to the recommended manufacturer’s protocol. The quality of isolated RNA was verified with electrophoresis on 2% agarose gel. Quality and quantity of the RNA was measured with spectrophotometric analysis (NanoDropND-1000, Thermo Scientific, Waltham, MA, USA). Reverse transcription was performed using High-Capacity cDNA KIT with RNAse inhibitor (Applied Biosystems, Foster City, CA, USA). SYBR Green detection (SYBR Select Master Mix, Life Technologies, Carlsbad, CA, USA) was used to perform quantitative RT-PCR on StepOnePlus^®^ Real-Time PCR System (Life Technologies) according to manufacturer’s instructions. Primers (Sigma-Aldrich) used for amplification of genes of interest are listed in Table S3. All primers were verified to yield a single PCR product with the correct molecular weight, and the absence of signal was verified when reverse transcription was omitted. The results were normalized to the expressions of endogenous reference genes (HPRT1, hypoxanthine phosphoribosyltransferase 1; B2M, beta-2-microglobulin). Experimental data can be found in Table S4.

### 2.3. Statistical Analysis

PCR efficacy and quantification cycle values for each sample were determined with LinRegPCR software (version 2017.1). Shapiro-Wilk test was used as normality test. To compare two groups with normally distributed data, Student’s *t*-test was used. For non-normally distributed data, we performed the Mann-Whitney test. ANOVA was used to assess the differences among multiple groups. Statistical correlation between two data groups was determined by Pearson correlation coefficients. All data were handled by GraphPad Prism (GraphPad Software Inc., San Diego, CA, USA, version 6). All data are presented as average ± standard error of the mean.

## 3. Results

### 3.1. Clinical and Biochemical Characteristics of Patients with Heart Failure

The wet weight of the explanted hearts was higher than the physiologic range (250–350 g) and this was observed both in the sex-specific group of women and men. The diagnosed HF was in last stages as shown by the left ventricle ejection fraction (LVEF) that was decreased to 22.64 ± 1.407%. The failing hearts of women displayed higher ejection fraction than male hearts. Both average Left ventricle end-diastolic diameter (LVEDD) and right ventricle end-diastolic diameter (RVEDD) was increased. QT interval values were within the physiological range (<0.44 s) and QRS complex values were slightly elevated (Table 1).

The average age of patients was 50.84 years, where women were insignificantly younger than men. The patients’ average BMI fell within the overweight range. Blood pressure values were within the physiological limits, although the heart rate was elevated (Table 1).

Table 1 also summarizes the biochemical parameters of the patient group. Plasmatic concentrations of N-terminal pro B-type natriuretic peptide (NT-proBNP) and troponin T were in the range of values that confirms end-stage HF. We detected no sex-specific differences in these concentrations. Levels of cholesterol and triacylglyceride (TAG) were not increased, possibly due to antihyperlipidemic therapy.

### 3.2. Expression of TRPC Channels

**Figure 1:**
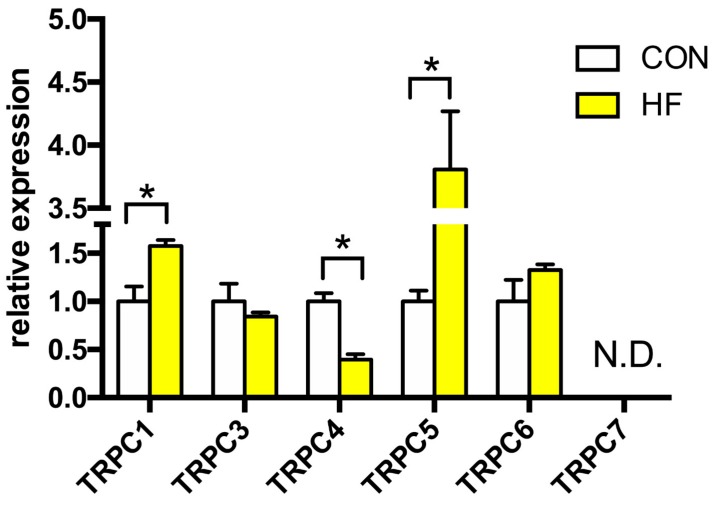
Relative gene expression of TRPC channels in the left ventricle of patients with heart failure (HF, n = 43) and non-failing (CON, n = 5) controls. N.D.—Non-detectable. Student’s *t*-test: * *p* < 0.05 vs. CON.

We detected the expression of TRPC1, −3, −4, −5 and −6 channels, whereas TRPC7 was not detectable (Figure 1). The expression of *Trpc1* gene was increased by more than 50% in the failing myocardium. The strongest elevation in expression, 3.8 times higher in failing hearts compared to CON, was observed in TRPC5 channel. Gene expression of TRPC4 channel was downregulated by 60%. There was no change between the groups in TRPC3 and TRPC6 channels expression.

### 3.3. Expression of TRPM Channels

**Figure 2:**
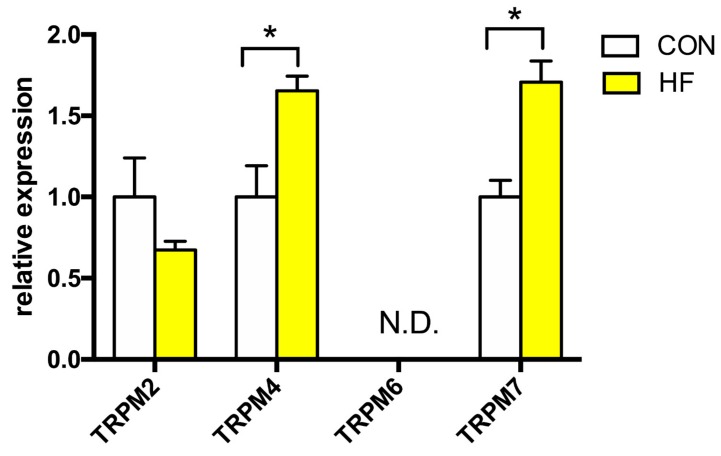
Relative gene expression of TRPM channels in the left ventricle of patients with heart failure (HF, n = 43) and non-failing (CON, n = 5) controls. N.D.—Non-detectable. Student’s *t*-test: * *p* < 0.05 vs. CON.

To this date, to our knowledge there are no information that links the TRPM channels subfamily and human HF, and only sparse data is available about their expression in human heart in general. We studied the expression profile of TRPM2, −4, −6 and −7 (Figure 2). The expression of TRPM2 channel was not altered in HF, however, we detected significantly increased expression of TRPM4 (more than 65% compared to CON) and TRPM7 channels (about 170% of CON). TRPM6 channel expression was not detected in either CON or failing myocardium.

### 3.4. Expression of TRPV Channels

**Figure 3:**
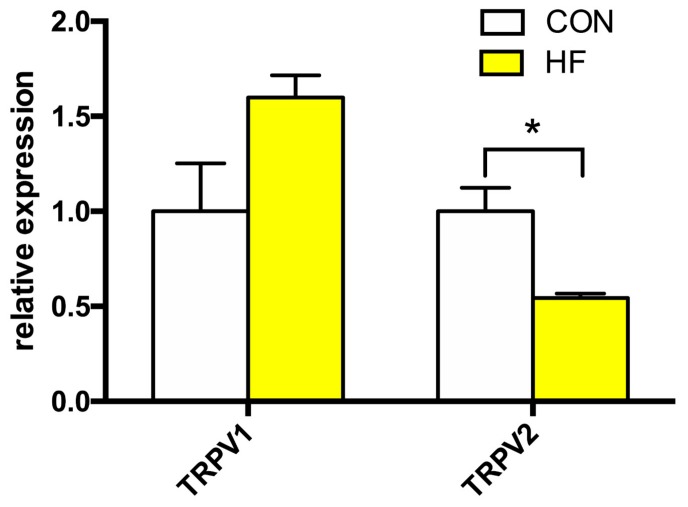
Relative gene expression of TRPV channels in the left ventricle of patients with heart failure (HF, n = 43) and non-failing (CON, n = 5) controls. N.D.—Non-detectable. Student’s *t*-test: * *p* < 0.05 vs. CON.

Analysis of the mRNA expression levels of TRPV1 and TRPV2 channels is summarized in Figure 3. We discovered significant decrease in gene expression levels of TRPV2. The average expression of TRPV1 displayed a tendency toward an increase in the HF group; however, this effect did not reach statistical significance.

### 3.5. The Expression of TRPC1 Correlates with MEF2c Gene Expression

**Figure 4:**
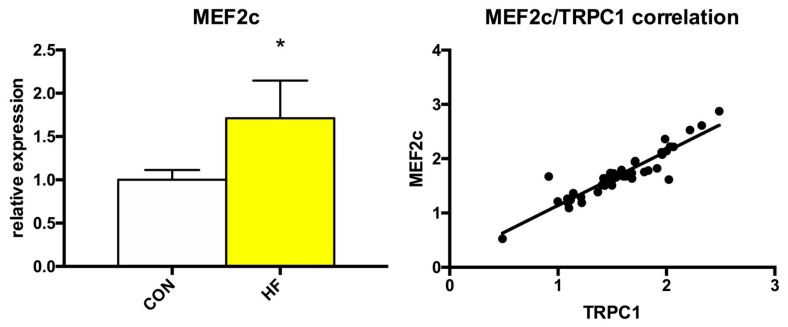
Relative gene expression of MEF2c and positive correlation between MEF2c and TRPC1 relative expressions. Student’s *t*-test: * *p* < 0.05 vs. CON; Pearson R^2^ = 0.8487.

As showed above, the expression of several TRP channels was significantly changed in failing myocardium. Because the alterations to Ca^2+^ homeostasis could affect some Ca^2+^-dependent transcription factors, we also analyzed the expression of MEF2a, MEF2c and NFAT3. While we detected no significant changes in the expression of MEF2a or NFAT3 (Table S1), MEF2c expression was significantly elevated by more than 70% and its expression positively correlated with *Trpc1* expression (Figure 4).

### 3.6. Clinical Status and TRP Expression

To better characterize the role of individual TRP channel isoforms in and their connection to different pathophysiological mechanisms of HF, we analyzed the gene expressions in regard to different subgroups or correlation with different parameters. Interestingly, we did not detect any significant differences in TRP expression between male and female HF patients, nor between HF based on ischemic or non-ischemic background. Furthermore, the *Trp* gene expression was not influenced by (i.e., no significant correlation was detected with) either functional heart parameters (QT, QRS duration, LVEF), clinical parameters (age, BMI, SBP, DBP or HR), nor biochemical data (NT-proBNP, troponin T levels, total cholesterol or TAG) (Table S2).

## 4. Discussion

HF is a complex syndrome defined as a progressive loss of heart’s blood-pumping function. HF invariably leads to cardiac hypertrophy and progressive disruption of calcium homeostasis [3,4]. TRP channels are non-voltage dependent, calcium-permeable cation channels that have been shown to play a critical role in HF and myocardial hypertrophy development [14]. However, there is limited data about their expression in a failing human heart. In this study, we focused on determining the gene expression changes of channels from TRPC, TRPM and TRPV subfamily in human HF compared to non-failing healthy controls.

### 4.1. Clinical and Biochemical Data

Clinical and biochemical data received from outpatient documentation showed severe heart damage. According to National Institute for Health and Care Excellence, patients with NT-proBNP, one of the main clinical diagnostic biomarkers of HF, levels above 2000 ng/L are suspected for HF and need echocardiographic examination [21]. Average NT-proBNP and troponin C levels of HF patients included in this study were significantly increased. Together with the very low average LVEF these findings confirm the end-stage of HF. HF patients had much higher heart weight than the expected physiological values, probably due to cardiac hypertrophy development. As expected, in the male sex-specific group, the heart weight was significantly higher compared to women.

Interestingly, we detected no significant correlation between either clinical, functional or biochemical parameters of HF patients and the expression of followed genes. This might be explained by the end-stage of the heart failing (requiring heart transplant due to compensatory mechanisms reaching their limits). This would require follow-up of this question in earlier stages of HF.

### 4.2. TRPC Channels

TRPC channels are divided according their sequence homology and function into two subgroups: TRPC3/6/7 and TRPC1/4/5; TRPC2 is a pseudogene in humans [22]. The critical role of TRPC in cardiac hypertrophy and HF was shown in multiple animal models. In models of pressure overload induced- and norepinephrine induced hypertrophy, mouse lacking *Trpc1* gene were protected against hypertrophy development [23]. Although Bush et al. did not detect any change in gene expression of TRPC1 in human failing heart, Morine et al. showed significant increase in *Trpc1* gene expression, in agreement with our results [15,20]. Increased expression of TRPC1 channel can result in higher SOCE into the cardiomyocyte, where this calcium influx could activate several prohypertrophic signaling pathways [24]. Also, we found that *TRPC1* gene expression strongly correlates with expression of the MEF2c transcription factor. This could present one of mechanisms of gradual decay in the cardiomyocyte function, even though it was shown in animal models that TRPC-dependent Ca^2+^ current activates predominantly the calcineurin/NFAT pathway [16]. In our study, gene expression of TRPC4 channel was significantly decreased, which is controversial, because transgenic mice overexpressing dominant negative TRPC4 mouse showed significantly reduced cardiac hypertrophy after transverse aortic constriction [25]. In previous studies on human HF patients, TRPC4 channel gene expression was increased [20] or not changed [15]. This differences in reported *Trpc4* gene expression could be caused by different heterotetramers that TRPC4 can form [26] or the etiology of HF. Last member of TRPC family whose expression was significantly changed is TRPC5 channel. Although the TRPC5 channel was not detected in two previous studies, we found almost four times higher gene expression compared to the healthy group. TRPC5 is a redox sensitive channel. Oxidized phospholipids can activate this channel and cause acute stimulatory effects on Ca^2+^-entry that can be inhibited by TRPC5 antibody or dominant-negative mutant TRPC5 [27]. Nevertheless, TRPC5 is not often linked with cardiac hypertrophy or HF and more data is needed for elucidating its function in this pathology. Interestingly, members of TRPC family that are most under debate in association with hypertrophy and HF, TRPC3 and TRPC6, were not significantly changed in human setting. Interestingly, the stretch-activated TRPC6 seems to be also the key component in stress-stimulated contractility [28], possibly due to an increase in SR calcium load. This provides another Ca^2+^-dependent regulatory mechanism (independent of NFAT activation), which may be important in HF.

### 4.3. TRPM Channels

The TRPM subfamily contains eight isoforms. Selectivity to Ca^2+^ is different among them—TRPM7 is most selective for Ca^2+^ while TRPM4 and TRPM5 are virtually impermeable for Ca^2+^, although these two channels are Ca^2+^-activated [29,30,31]. To this date, the role of TRPM channels in human HF is unclear. To our knowledge, there is only one study that has investigated mRNA expression levels of TRPM channels in human HF. Morine et al. reported that the gene expressions of TRPM2, −3 and −8 were reduced in failing left ventricle (LV) and TRPM1, −6 and −7 were not detectable [20]. Our results showed increased gene expression of TRPM4 and −7 and a trend towards reduction of TRPM2 mRNA expression. TRPM6 channel was not detected. Increased mRNA expression of TRPM4 was detected in spontaneously hypertensive rats, a model of cardiac hypertrophy [32]. TRPM4 channel is Ca^2+^-activated, Na^+^- and K^+^-permeable and its increased expression can probably participate in development of delayed after-depolarizations (DADs) during cardiac hypertrophy [33]. We hypothesize that TRPM4 could participate in arrhythmia development during HF, but there is a need for further studies to confirm this hypothesis, because so far, the Ca^2^+-dependent transient inward current that is necessary for DADs development was detected only in human atrial cells and not in ventricular cells [34]. The role of TRPM4 in cardiac hypertrophy is controversial and it seems to work as a negative regulator of cardiac hypertrophy development after angiotensin II infusion [35]. Increased expression of TRPM7 during HF could result in fibrogenesis in LV. Ca^2+^ currents through TRPM7 in human atrial fibroblasts can mediate fibrogenesis together with TGF-β1 [36].

### 4.4. TRPV Channels

In this study, we analyzed the mRNA expression of two members of TRPV family—TRPV1 and TRPV2. *Trpv1* gene expression was not changed and gene expression of *Trpv2* was significantly reduced in HF. The TRPV2 channel is Ca^2+^-permeable channel and under basal conditions, it is localized both intracellularly [37] and at the intercalated disc [17]. Translocation to plasma membrane occurs after activation by several receptor agonists, such as insulin-like growth factor or platelet-derived growth factor, or by mechanical stress [37,38]. The role of TRPV2 channel in maintaining a normal cardiac function in healthy hearts and HF development is different. On the one hand, temporary elimination of TRPV2 in the normal heart results in disruption of intercalated disc structure and in decline of the heart function [39]. On the other hand, the inhibition of TRPV2 translocation to the plasma membrane improved myocardial dysfunction, improved survival of mice with dilated cardiomyopathy and slowed the progression of dilated cardiomyopathy [17]. In addition, in previous study, the *Trpv2* gene expression was increased in left ventricle in human HF [20] which indicates a more complex role of TRPV2 channel in human HF.

The body of data about involvement of TRP channels in the pathophysiology of HF is growing rapidly. However, published results from animal models or human samples are controversial and many important questions remain unresolved. The common theme seems to be a complex regulation and functional interplay between TRP isoforms. The interpretation of such data is further complicated by the fact that TRPs undergo different post-translational modifications, including phosphorylation, *N*-glycosylation or polyester modification or covalent modifications of cysteine residues.

### 4.5. Study Limitations

The main limitation of the study is the reliance on mRNA levels to assess the expressional patterns of TRP genes. The results presented here need to be expanded with data on protein expressions, posttranslational modifications, and function, which should provide deeper insight into the role of TRP in HF. Another limitation are generally low expression levels of genes of interest, both in CON and HF group. This contributes to a relatively high variability, which is already inherently high in clinical setting. This makes the interpretation of the correlation analysis particularly complicated and a targeted investigation should be performed in the near future. The same complication extends to the TRPC1/MEF2c interplay. Even though a similar relationship between TRPC1 and MEF2 was observed in skeletal muscle [40], a targeted approach to study this possible mechanism in cardiac tissue is warranted and required. Finally, end-stage HF represents a time point, where most of the damage to the heart is done, and similar analysis of earlier stages of human HF would help to identify the underlying pathophysiological mechanisms.

## 5. Conclusions

In conclusion, TRP channels play a key role in human HF development and underlie a complex, but distinct changes in their expression which often differs from that observed in animal models. We suggest that there is a crucial role of TRPC (TRPC1, TRPC4 and TRPC5), TRPM4 and −7, and TRPV2 channels. We also submit that if TRP channels should present a feasible pharmacological target for patients with HF, there is a need for investigation to further elucidate the role of TRP channels in man, and to investigate whether TRP channels are potential pharmacological targets in patients with HF.

## Figures and Tables

**Table 1:** Patients’ clinical characteristics.

	ALL	Female	Male
**HW** [g]	635.8 ± 24.09 (30)	415.8 ± 43.04 (4)	669.7 ± 19.97# (26)
**LVEF** [%]	22.64 ± 1.407 (42)	30.88 ± 5.363 (8)	20.71 ± 1.003 (34)
**LVEDD** [mm]	69.35 ± 1.750 (40)	54.50 ± 3.718 (8)	73.06 ± 1.346# (32)
**RVEDD** [mm]	33.87 ± 0.7404 (39)	32.25 ± 1.146 (8)	34.29 ± 0.8752 (31)
**QRS** [s]	0.1372 ± 0.006557 (36)	0. 1420 ± 0.01718 (7)	0. 1361± 0.007155 (27)
**QT** [s]	0, 4221 ± 0.00722 (34)	0.4263 ± 0.01225 (7)	0.4210 ± 0.008612 (27)
**Age** [year]	50.84 ± 1.281 (43)	46.88 ± 2.553 (8)	51.74 ± 1.433 (35)
**BMI** [kg/m^2^]	27.11 ± 0.6984 (42)	24.81 ± 1.797 (8)	27.65 ± 0.7356# (34)
**SBP** [mmHg]	109.3 ± 1.964 (42)	103.8 ± 5.775 (8)	110.6 ± 1.996 (34)
**DBP** [mmHg]	70.93 ± 1.194 (42)	69.00 ± 2.790 (8)	71.38 ± 1.330 (34)
**HR** [min^−1^]	79.95 ± 1.754 (43)	78.88 ± 2.689 (8)	80.21 ± 2.085 (34)
**NT-proBNP** [ng/l]	4666 ± 631.5 (40)	5132 ± 1152 (8)	4549 ± 741.7 (32)
**Troponin T** [ng/l]	30.18 ± 5.202 (31)	38.53 ± 17.99 (6)	28.09 ± 4.911 (25)
**Cholesterol** [mmol/l]	4.133 ± 0.1892 (32)	4.528 ± 0.3372 (5)	4.059 ± 0.2145 (27)
**TAG** [mmol/l]	1.684 ± 0.2430 (31)	1.095 ± 0.1395 (6)	1.825 ± 0.2936# (25)

HW—Heart weight, LVEF—Left ventricle ejection fraction, LVEDD—Left ventricle end-diastolic diameter, RVEDD—Right ventricle end-diastolic diameter, BMI—Body mass index, SBP—Systolic blood pressure, DBP—Diastolic blood pressure, HR—Heart rate, NT-proBNP—N-terminal pro B-type natriuretic peptide and TAG—triacylglyceride blood concentrations. Number of patients where data was available shown in brackets; Student’s *t*-test: # *p* < 0,05 vs. F.

## Supplementary Materials

The following are available online at https://www.mdpi.com/1010-660X/55/7/380/s1, Table S1: Relative expression of MEF2a, MEF2c and NFAT3; Table S2: Correlation analysis; Table S3: Primer sequences; Table S4: Experimental data.
