# Supplementary material for: TRP Channels Expression Profile in Human End-Stage Heart Failure
Source: Medicina (Kaunas). 2019 Jul 16;55(7):380. doi: 10.3390/medicina55070380 (PMC6681334; doi:10.3390/medicina55070380)
Supplement: Supplementary file 1 [file medicina-55-00380-s001.zip › Table S1 + S2 + S3 revised.docx]

**Table S1:** Relative expression of MEF2a, MEF2c and NFAT3 in the left ventricle of patients with heart failure (HF, n = 43) and non-failing (CON, n = 5) controls. Student’s t-test: * p < 0,05 vs. CON.

|  | **n** | **MEF2a** | **MEF2c** | **NFAT3** |
| --- | --- | --- | --- | --- |
| **CTR** | 5 | 1.000±0.137 | 1.000±0.045 | 1.000±0.169 |
| **HF** | 43 | 1.180±0.047 | 1.711±0.066* | 0.972±0.041 |

**Table S2:** Correlation analysis of gene expression and the patients’ physiological and biochemical characteristics. Shown is the coeficient of determination R^2^ (*p<0.05).

|  | **Age** | **BMI** | **SBP** | **DBP** | **HR** | **NT-proBNP** | **Troponin T** | **Cholesterol** | **TAG** |
| --- | --- | --- | --- | --- | --- | --- | --- | --- | --- |
| **TRPC1** | 0,025 | 0,001 | 0,003 | 0,009 | 0,018 | 0,094 | 0,028 | 0,015 | 0,002 |
| **TRPC3** | 0,007 | 0,001 | 0,028 | 0,034 | 0,039 | 0,002 | 0,027 | 0,007 | 0,035 |
| **TRPC4** | 0,018 | 0,000 | 0,069 | 0,000 | 0,005 | 0,023 | 0,036 | 0,008 | 0,002 |
| **TRPC5** | 0,065 | 0,002 | 0,047 | 0,04 | 0,000 | 0,061 | 0,002 | 0,19 | 0,046 |
| **TRPC6** | 0,005 | 0,013 | 0,004 | 0,01 | 0,034 | 0,001 | 0,013 | 0,008 | 0,014 |
| **TRPM2** | 0,074 | 0,008 | 0,002 | 0,051 | 0,009 | 0,001 | 0,026 | 0,036 | 0,08 |
| **TRPM4** | 0,003 | 0,142 | 0,001 | 0,000 | 0,039 | 0,003 | 0,076 | 0,115 | 0,153 |
| **TRPM7** | 0,005 | 0,048 | 0,008 | 0,006 | 0,005 | 0,065 | 0,008 | 0,172 | 0,211 |
| **TRPV1** | 0,014 | 0,000 | 0,034 | 0,058 | 0,011 | 0,000 | 0,002 | 0,001 | 0,038 |
| **TRPV2** | 0,001 | 0,029 | 0,008 | 0,001 | 0,01 | 0,001 | 0,016 | 0,154 | 0,078 |

Table S3*.* Primer sequences used for amplification in qRT-PCR of housekeeping genes (B2M, HPRT1) and of the genes of interest.

| **Official gene symbol** | ***Official Gene Name***  **Primer Sequences (5’ → 3’)** |
| --- | --- |
| **B2M** | *beta-2-microglobulin*  Forward: TCCGTGGCCTTAGCTGTGCTT  Reverse: TCCATTCTCTGCTGGATGACGTGAG |
| **HPRT1** | *hypoxanthine phosphoribosyltransferase 1*  Forward: AGCCCTGGCGTCGTGATTAGTGA  Reverse: GGTCACAATGTGATGGCCTCCCA |
| **MEF2c** | *myocyte enhancer factor 2C*  Forward: TCCATCCCAGTGTCCAGCCACA  Reverse: GCCTGCACCAGACGTGAGGT |
| **TRPC1** | *transient receptor potential cation channel subfamily C member 1*  Forward: CTATGGAGAAGAACTGCAGTC  Reverse: GGAAGTCAAGTAACGATGCAC |
| **TRPC3** | *transient receptor potential cation channel subfamily C member 3*  Forward: TGTTCAATGCCTCAGACAGG  Reverse: AGTGTCACTTCACTGAGGTC |
| **TRPC4** | *transient receptor potential cation channel subfamily C member 4*  Forward: TGGAACAATAGGGAGGCGAG  Reverse: TCTCTCAAGTGGTCCTGCAG |
| **TRPC5** | *transient receptor potential cation channel subfamily C member 5*  Forward: TGGAGAAGAGCAAAATGGAGGG  Reverse: TTTTCGGACAACCTCTTGCCA |
| **TRPC6** | *transient receptor potential cation channel subfamily C member 6*  Forward: CTTTTGCTGAAGGCAAGAGG  Reverse: CTGCACAGATCAAGGAGTCC |
| **TRPM2** | *transient receptor potential cation channel subfamily M member 2*  Forward: GTCAAGCTCAACGTGCAGG  Reverse: ATTGTCCACGGAGAGCTGG |
| **TRPM4** | *transient receptor potential cation channel subfamily M member 4*  Forward: TGCGCGCCGAGATGTAT  Reverse: AAAGAAGCAGGTCGCTCCAG |
| **TRPM7** | *transient receptor potential cation channel subfamily M member 7*  Forward: CCTGTGCTTTTGATCTCCTGTC  Reverse: AACAGACAGCCCATATTGCCC |
| **TRPV1** | *transient receptor potential cation channel subfamily V member 1*  Forward: CTTCGAGTAGCAACCGCCTT  Reverse: CCCAGTGTGCAACCAGCTA |
| **TRPV2** | *transient receptor potential cation channel subfamily V member 2*  Forward: CAGCGGGAGTTTTCAGGACT  Reverse: GCCTGCTTCTTCAGGGTAGG |
