# Supplementary material for: TRP Channels Expression Profile in Human End-Stage Heart Failure
Source: Medicina (Kaunas). 2019 Jul 16;55(7):380. doi: 10.3390/medicina55070380 (PMC6681334; doi:10.3390/medicina55070380)
Supplement: Supplementary file 1 [file medicina-55-00380-s001.zip › Table S4 revised.pdf]

Group	Sample Nr.	TRPC1		TRPC3		TRPC4		TRPC5		TRPC6		TRPM2	
		C_T	expression	C_T	expression	C_T	expression	C_T	expression	C_T	expression	C_T	expression
	01	32,474	0,504	33,691	0,798	34,239	1,259	32,704	1,345	35,996	0,703	33,979	0,685
	02	31,023	0,788	33,222	0,688	33,936	0,964	32,842	0,665	33,302	1,803	32,700	0,992
**CTR**	03	30,759	1,288	33,160	0,995	35,067	0,733	32,550	0,906	35,996	0,639	33,262	0,974
	04	30,993	1,258	32,446	1,715	34,529	1,100	32,951	1,064	35,041	1,168	32,399	1,879
	05	31,501	1,162	34,094	0,804	35,238	0,944	33,415	1,021	36,534	0,686	34,983	0,469
	01	31,432	1,961	33,900	1,462	50,364	0,000	31,968	3,130	34,734	2,774	34,593	0,969
	02	32,072	1,090	34,291	0,947	38,849	0,180	31,865	3,110	35,949	1,223	34,066	1,102
	03	32,337	1,101	34,365	1,074	38,183	0,305	30,681	7,908	36,507	1,092	34,526	0,980
	04	31,432	1,513	34,174	0,958	39,071	0,151	31,737	2,965	35,294	1,610	34,756	0,676
	05	31,710	1,406	35,242	0,556	45,819	0,004	31,347	3,860	36,118	1,164	35,280	0,535
	06	31,841	1,467	34,644	0,897	39,162	0,178	30,165	10,715	36,077	1,342	35,257	0,613
	07	31,610	1,796	35,368	0,620	38,628	0,253	31,198	6,010	36,145	1,380	34,313	1,178
	08	32,418	0,998	35,258	0,600	38,330	0,268	30,531	8,593	36,297	1,157	35,212	0,608
	09	31,220	1,431	34,754	0,563	37,803	0,248	31,756	2,601	35,673	1,105	34,411	0,697
	10	30,763	1,682	33,576	1,016	36,246	0,509	30,025	6,563	35,354	1,159	34,297	0,668
	11	31,620	1,537	35,119	0,619	38,616	0,219	29,817	11,650	36,145	1,188	35,632	0,445
	12	31,795	1,483	34,505	0,959	36,922	0,582	29,639	13,312	35,536	1,737	34,528	0,950
	13	31,163	1,583	34,405	0,742	38,673	0,166	31,717	2,728	35,330	1,407	34,993	0,518
	14	31,093	1,116	35,085	0,334	38,652	0,113	32,067	2,105	35,479	0,881	33,008	1,209
	15	31,939	0,487	33,395	0,667	37,995	0,117	33,349	0,821	35,751	0,558	32,003	1,649
	16	32,294	0,914	35,402	0,468	39,356	0,131	32,112	2,516	35,936	1,182	35,989	0,318
	17	31,375	1,503	34,523	0,746	40,062	0,085	31,734	3,017	35,510	1,384	35,167	0,501
	18	31,213	1,508	34,370	0,744	37,486	0,308	32,158	2,196	36,297	0,844	34,459	0,710
	19	31,809	1,368	34,979	0,672	38,019	0,301	31,240	4,480	36,186	1,161	35,391	0,516
	20	31,420	1,497	33,982	1,055	35,090	1,253	32,875	1,602	35,281	1,591	36,303	0,252
	21	31,077	1,988	34,755	0,717	36,727	0,562	31,282	4,102	36,088	1,140	36,272	0,278
**HF**	22	31,304	1,709	34,982	0,618	37,156	0,440	31,445	4,242	35,175	1,788	37,177	0,156
	23	31,693	1,141	36,040	0,277	34,156	1,862	31,493	3,100	35,980	1,003	35,026	0,504
	24	31,232	1,681	34,345	0,851	39,354	0,127	31,882	2,601	35,079	1,768	34,800	0,647
	25	31,891	1,213	35,572	0,439	36,221	0,736	32,359	2,257	35,079	1,901	37,207	0,155
	26	30,762	2,217	33,787	1,180	36,999	0,448	31,484	3,219	36,085	1,053	34,951	0,584
	27	31,012	1,832	33,694	1,198	39,907	0,090	31,184	4,249	35,263	1,536	35,755	0,340
	28	31,282	1,625	34,161	0,947	36,302	0,653	30,055	8,498	35,296	1,578	34,575	0,742
	29	30,241	2,003	33,154	1,136	37,405	0,238	31,480	2,261	34,698	1,406	32,577	1,699
	30	30,556	2,023	32,948	1,569	37,377	0,294	29,825	7,055	34,759	1,664	34,719	0,544
	31	31,794	1,085	34,556	0,681	37,040	0,400	34,090	0,715	35,703	1,168	34,346	0,779
	32	30,596	1,603	33,568	0,880	37,179	0,266	33,360	0,709	34,807	1,319	34,218	0,605
	33	31,135	1,713	34,420	0,782	38,495	0,194	32,732	1,650	34,807	1,951	34,804	0,620
	34	30,440	1,914	33,389	1,063	37,431	0,252	32,336	1,364	35,413	1,052	34,836	0,446
	35	30,490	2,325	33,539	1,217	38,799	0,151	32,583	1,669	35,478	1,275	35,652	0,335
	36	30,112	2,488	33,854	0,858	36,462	0,453	32,952	1,068	35,681	0,979	33,805	0,906
	37	30,961	1,955	34,283	0,872	36,594	0,553	32,134	2,245	35,556	1,369	35,168	0,507
	38	30,327	2,065	33,432	1,044	34,646	1,134	32,388	1,341	34,424	1,754	33,748	0,887
	39	30,566	2,034	34,119	0,789	36,543	0,466	32,991	1,160	35,792	0,996	35,260	0,393
	40	31,144	1,419	33,715	0,993	36,445	0,486	31,441	3,012	35,893	0,936	33,946	0,883
	41	31,278	1,586	34,512	0,747	37,445	0,344	32,353	2,118	35,893	1,134	33,968	1,055
	42	32,077	1,219	34,032	1,240	36,900	0,575	33,434	1,801	35,698	1,560	34,858	0,754
	43	31,970	1,490	35,866	0,475	36,227	0,945	32,096	3,425	37,297	0,793	38,733	0,077

target gene (continued)

Group	Sample Nr.	TRPM4		TRPM7		TRPV1		TRPV2		MEF2a		MEF2c		NFAT3	
		C_T	expression	C_T	expression	C_T	expression	C_T	expression	C_T	expression	C_T	expression	C_T	expression
	01	29,371	1,556	32,674	1,329	34,100	1,508	31,971	0,829	28,148	0,714	31,350	0,927	27,911	1,737
	02	30,080	0,545	32,699	0,705	34,359	0,697	30,429	1,249	27,387	0,736	30,720	0,883	28,220	0,938
**CTR**	03	29,183	1,072	32,150	1,049	34,399	0,779	30,765	1,142	27,508	0,955	30,867	1,127	28,892	0,872
	04	29,609	1,237	33,056	1,017	36,482	0,348	31,309	1,185	27,565	1,037	31,079	1,115	29,299	0,766
	05	31,229	0,590	33,807	0,900	34,185	1,669	32,692	0,595	27,268	1,558	31,723	0,948	29,856	0,687
	01	29,541	2,105	33,840	1,141	33,857	2,600	33,650	0,404	28,139	1,490	31,224	2,074	31,182	0,499
	02	29,691	1,796	33,191	1,453	34,677	1,510	32,904	0,622	28,696	0,870	31,813	1,189	30,302	0,695
	03	29,482	2,546	32,454	2,583	34,914	1,646	32,901	0,779	29,059	0,828	32,238	1,091	30,438	0,758
	04	30,295	1,102	33,416	1,153	35,068	1,063	32,734	0,615	28,128	1,157	31,099	1,727	29,551	1,032
	05	28,939	2,572	32,693	1,677	35,261	0,979	33,173	0,473	28,323	1,131	31,471	1,516	29,953	0,889
	06	29,777	2,120	32,067	3,092	33,922	2,910	33,187	0,640	28,571	1,099	31,524	1,658	30,244	0,842
	07	29,600	2,458	32,668	2,417	35,499	1,214	33,184	0,668	28,326	1,357	31,535	1,754	29,819	1,160
	08	30,704	1,211	33,124	1,864	34,806	1,741	33,396	0,556	29,289	0,686	31,983	1,211	30,717	0,610
	09	29,345	1,733	32,981	1,258	35,590	0,696	32,322	0,719	28,015	1,031	31,022	1,505	29,466	0,904
	10	29,673	1,272	31,652	2,123	33,300	2,351	32,519	0,563	27,600	1,182	30,698	1,634	28,902	1,134
	11	29,250	2,572	31,017	4,504	34,346	2,008	32,840	0,712	28,255	1,219	31,379	1,659	29,975	0,909
	12	29,167	2,778	31,486	3,697	34,679	1,700	32,794	0,754	28,573	1,079	31,419	1,736	30,018	0,949
	13	29,977	1,213	33,394	1,058	34,196	1,601	33,719	0,289	27,882	1,195	30,886	1,748	29,612	0,885
	14	29,776	1,304	32,321	1,682	34,323	1,416	32,774	0,518	28,090	0,711	30,802	1,241	29,301	0,721
	15	29,886	1,026	33,384	0,852	35,229	0,705	32,305	0,596	28,624	0,375	31,701	0,524	30,242	0,297
	16	30,748	0,890	33,814	1,012	35,314	0,979	33,596	0,367	29,025	0,684	31,182	1,671	30,726	0,516
	17	29,820	1,490	32,609	1,721	34,310	1,676	33,010	0,519	28,040	1,171	31,143	1,614	29,525	1,006
	18	28,936	2,383	32,686	1,556	33,505	2,507	32,664	0,614	27,903	1,158	30,874	1,728	29,527	0,914
	19	29,510	1,983	31,808	2,784	35,010	1,233	33,540	0,402	28,554	1,016	31,674	1,385	29,954	0,919
	20	31,433	0,592	34,603	0,699	34,488	1,590	33,550	0,381	28,053	1,189	31,294	1,507	29,710	0,921
	21	30,062	1,335	32,803	1,626	34,836	1,280	33,635	0,355	27,966	1,352	30,678	2,361	29,221	1,335
**HF**	22	30,466	1,193	33,755	1,177	35,226	1,164	33,396	0,474	28,192	1,164	30,977	1,943	29,706	0,982
	23	29,855	1,297	32,895	1,335	36,223	0,491	32,910	0,494	28,510	0,811	31,284	1,364	29,900	0,738
	24	29,532	1,670	33,493	1,063	36,104	0,558	32,914	0,522	28,009	1,224	31,065	1,734	29,547	1,018
	25	30,641	0,988	33,608	1,162	35,773	0,780	33,790	0,335	28,613	0,913	31,674	1,291	30,030	0,817
	26	29,862	1,334	32,992	1,317	33,871	1,985	33,375	0,374	27,434	1,721	30,432	2,527	29,074	1,346
	27	30,276	1,146	32,733	1,643	33,528	2,671	33,228	0,455	27,612	1,485	30,944	1,781	29,363	1,085
	28	29,741	1,609	31,580	2,896	35,486	0,872	33,589	0,363	27,862	1,333	31,119	1,674	29,598	0,984
	29	28,260	2,458	31,712	1,700	33,303	1,934	31,778	0,762	26,897	1,570	30,017	2,142	28,488	1,265
	30	29,095	1,719	31,832	1,857	32,977	2,704	32,021	0,750	27,182	1,614	30,816	1,614	28,765	1,307
	31	29,512	1,745	34,305	0,744	33,512	2,587	32,948	0,526	28,554	0,798	31,431	1,262	30,163	0,636
	32	28,457	2,111	33,418	0,728	34,527	0,920	32,334	0,508	27,214	1,286	30,410	1,675	28,933	0,959
	33	29,040	2,372	33,399	1,174	33,197	3,177	32,468	0,742	28,010	1,175	30,801	1,955	29,410	1,063
	34	29,180	1,422	32,761	1,038	34,334	1,072	31,960	0,680	27,162	1,440	30,408	1,821	28,654	1,233
	35	30,178	1,104	33,863	0,870	34,311	1,540	32,772	0,560	27,341	1,619	30,183	2,612	28,588	1,606
	36	28,676	2,183	32,844	1,130	33,939	1,526	32,268	0,627	26,756	1,965	29,760	2,874	28,445	1,492
	37	30,370	1,011	32,675	1,576	33,842	2,077	34,106	0,236	27,778	1,389	30,714	2,118	29,186	1,250
	38	29,394	1,267	31,912	1,579	32,476	3,192	32,586	0,454	27,109	1,500	30,094	2,219	28,655	1,242
	39	29,774	1,250	33,412	0,957	34,390	1,306	32,707	0,519	27,448	1,391	30,307	2,222	28,766	1,322
	40	28,624	2,568	31,547	2,393	33,374	2,415	32,457	0,629	27,867	1,068	30,792	1,639	29,381	0,901
	41	29,535	1,800	32,666	1,705	35,435	0,888	32,523	0,732	28,015	1,183	30,963	1,791	29,525	1,001
	42	30,296	1,849	33,371	1,978	35,627	1,291	33,069	0,825	28,836	0,897	32,005	1,188	30,306	0,778
	43	31,981	0,570	31,866	3,458	38,248	0,241	34,480	0,274	28,539	1,231	31,735	1,603	30,328	0,879

clinical and biochemical characteristics / *characteristics of explanted heart*

Group	Sample Nr.	Age	BMI	SBP	DBP	HR	NT-proBNP	Troponin T	Cholesterol	TAG	*gender*	HW	LVEF	LVEDD	RVEDD	QRS	QT	*HF type*
		[y]	[kg/m2]	[mmHg]	[mmHg]	[min-1]	[ng/l]	[ng/l]	[mmol/l]	[mmol/l]		[g]	[%]	[mm]	[mm]	[s]	[s]	
	01																	
	02																	
**CTR**	03																	
	04																	
	05																	
	01	58	25,260	104	72	100	10915	1700	2,3	0,71	*M*	670	10	68	39	0,114	0,306	DCM
	02	54	29,032	131	87	63	367	-	4,2	1,53	*M*	760	25	64	28	0,1	0,413	CAD
	03	62	31,745	110	75	74	2157	20	5,9	1,78	*M*	700	20	72	32	0,172	0,444	DCM
	04	57	20,529	109	62	52	3142	30	4,3	1,13	*M*	530	40	66	33	0,2	0,48	RCM
	05	51	39,792	96	71	83	9012	27	5,1	2,4	*M*	700	20	76	40	0,11	0,388	DCM
	06	52	25,100	114	70	73	4314	-	5,4	1,5	*M*	560	24	64	45	0,15	0,448	DCM
	07	57	32,327	90	65	84	547	14	5,4	8,18	*M*	672	23	69	30	0,128	0,466	DCM
	08	36	19,628	96	63	73	1789	-	5,3	1,37	*F*	336	45	59	32	0,11	0,448	RCM
	09	44	32,596	103	70	100	1434	95	2,2	1,23	*M*	-	25	68	34	0,169	0,411	CAD
	10	31	30,972	113	73	92	914,5	-	5,2	2,82	*M*	723	20	74	-	0,074	0,364	DCM
	11	41	24,535	95	65	75	3233	6	4,9	1,32	*M*	-	20	72	36	0,114	0,406	DCM
	12	40	23,323	89	76	90	2215	6	3,6	1,13	*F*	520	22	62	34	0,102	0,388	CAD
	13	50	23,225	95	59	94	5473	10	4,1	1,36	*M*	530	20	82	31	0,102	0,33	DCM
	14	50	24,691	98	73	72	19015	-	1,9	0,93	*M*	-	20	74	33	0,09	0,363	DCM
	15	54	26,556	107	73	85	1778	33	3,4	1,61	*M*	660	20	76	32	0,16	0,47	CAD
	16	62	24,977	130	85	80	-	9	3,9	2,1	*M*	440	20	-	-	0,166	0,454	CAD
	17	34	29,364	120	72	85	7861	3	-	-	*M*	660	15	-	-	0,118	0,458	DCM
	18	55	28,704	95	65	63	3031	29	4,8	1,95	*M*	593	-	-	-	0,13	0,415	DCM
	19	31	23,449	120	75	70	4279	11	2,4	0,58	*M*	680	10	82	42	0,146	0,417	DCM
	20	57	22,161	103	59	79	2869	17	4,1	1,45	*M*	576	35	61	33	0,16	0,422	AS
	21	53	22,959	100	60	83	6199	25	3,9	0,53	*F*	452	15	62	38	0,201	0,456	DCM
**HF**	22	49	21,908	85	60	80	5252	-	-	-	*F*	-	18	54	35	0,105	0,397	RCM
	23	55	28,082	135	65	74	2856	17	4,1	2,2	*M*	753	20	85	25	0,181	0,436	CAD
	24	53	30,116	130	76	93	764,1	27	3,7	1,19	*M*	760	15	68	39	0,12	0,461	DCM
	25	39	-	110	85	55	918,6	-	5,13	-	*M*	762	20	80	31	0,128	0,458	DCM
	26	58	27,852	135	80	70	6531	6	-	1,49	*F*	-	55	36	28	0,173	0,469	HCM
	27	40	28,406	110	75	83	1989	15	3,54	-	*M*	705	20	77	43	0,164	0,431	CAD
	28	55	25,359	99	62	95	-	-	-	-	*M*	687	20	75	31	-	0,385	CAD
	29	59	25,796	120	75	80	-	98,71	-	-	*M*	526	18	79	27	0,155	0,4	CAD
	30	42	23,795	110	75	86	11903	115,4	4,68	0,92	*F*	355	45	41	31	0,108	0,398	ARVC
	31	46	23,796	110	70	89	927,7	6,54	-	-	*M*	829	28	79	33	0,068	0,456	CAD
	32	53	20,148	105	55	71	6491	-	3,5	1,17	*M*	670	25	85	33	-	-	CAD
	33	51	31,354	-	-	-	6201	-	4,4	1,41	*M*	905	20	84	37	-	-	DCM
	34	50	22,993	98	65	69	2927	66,76	5,16	1,13	*F*	-	20	65	30	-	-	DCM
	35	47	36,052	117	73	80	4242	12	-	-	*F*	-	27	57	30	0,195	0,428	DCM
	36	60	24,057	105	75	78	11673	43,8	2,42	1,16	*M*	-	13	83	39	0,247	0,485	DCM
	37	59	27,472	109	72	68	3468	29,5	-	-	*M*	660	20	56	29	0,12	0,4	CAD
	38	59	30,323	125	85	79	3469	34	4,07	1,4	*M*	700	20	70	32	-	-	CAD
	39	62	23,939	103	62	101	11758	-	3,65	1,14	*M*	-	15	65	32	0,12	-	DCM
	40	54	35,986	123	76	84	677,7	-	5,59	3,38	*M*	-	20	74	31	-	-	CAD
	41	55	30,992	114	78	79	2581	30,7	-	-	*M*	-	25	62	40	0,14	-	DCM
	42	55	29,412	120	75	94	4705	23,7	-	-	*M*	-	18	78	34	-	-	DCM
	43	56	29,772	110	70	80	6752	44,3	-	-	*M*	-	20	70	39	0,1	-	DCM

Agenda:

BMI	*Body mass index*
SBP	*Systolic Blood Pressure*
DBP	*Diastolic Blood Pressure*
HR	*Heart Rate*
NT-proBNP	*N-terminal pro B-type natriuretic peptide*
TAG	*Triacylglycerides*
HW	*Heart Weight*
LVEF	*Left Ventricle Ejection Fraction*
LVEDD	*Left Ventricle End-diastolic Diameter*
RVEDD	*Right Ventricle End-diastolic Diameter*
